# Laparoscopic Hysteropexy: How, When and for Whom Is It an Alternative Option? A Narrative Review of the Literature

**DOI:** 10.3390/jcm14041080

**Published:** 2025-02-08

**Authors:** Anna Pitsillidi, Günter Karl Noé

**Affiliations:** 1 Department of OB/GYN, Rheinland Klinikum Dormagen, Dr.-Geldmacher-Straße 20, 41540 Dormagen, Germany; guenter-karl.noe@rheinlandklinikum.de; 2 Department of OB/GYN, University of Witten Herdecke, Dr.-Geldmacher-Straße 20, 41540 Dormagen, Germany

**Keywords:** hysteropexy, prolapse, laparoscopy, uterine preservation

## Abstract

**Background/Objectives**: Surgical repair of apical prolapse most commonly includes hysterectomy. However, nowadays, the number of women who seek uterine preserving surgical treatment is increasing. Our objective is to review the current evidence on laparoscopic hysteropexy techniques, outcomes and appropriate patient selection. **Methods**: A literature search was carried out in MEDLINE/PubMed and ClinicalTrials.gov databases. The search was restricted to humans, female patients and currently used surgical procedures. **Results**: Laparoscopic hysteropexy was found to be associated with good anatomic outcomes, symptom improvement and low complication or reoperation rates. **Conclusions**: Laparoscopic hysteropexy appears to be a good alternative option for women who undergo surgical treatment for apical prolapse and desire preservation of the uterus. However, further prospective comparative studies, as well as longer follow-up periods, are necessary for evaluating long-term safety and efficacy outcomes of the method.

## 1. Introduction

POP (pelvic organ prolapse) is a clinical condition in which the pelvic organs protrude or herniate through the vaginal walls and pelvic floor, affecting many women and their quality-of-life worldwide [1,2]. Support of the apical compartment seems to be really crucial for the restoration of the pelvic floor’s anatomy and function. The surgical treatment of apical prolapse consists of techniques using native tissue or meshes with a concomitant hysterectomy in order to achieve vaginal vault or cervical suspension. Hysterectomy alone is shown to be unable to correct the apical defect [3]. However, its role in the success of apical support is doubtful even while combined with other methods [4,5]. Nowadays, women have an increasing desire for uterus-preserving prolapse treatment. More specifically, it is shown that more than one-third of women will prefer uterine-preserving prolapse surgery, provided the outcomes are similar, and more than one-fifth of them still choose to preserve their uterus, even if it is associated with poorer outcomes. There are several reasons why women may make this decision, including the desire to preserve fertility, the belief that the uterus affects sexuality, and the latter’s connection to a woman’s sense of identity [6,7]. Several uterus-preserving techniques, abdominal, vaginal and laparoscopic, have been described and shown to have decreased operative times and estimated blood loss. Unfortunately, no consensus for choosing the appropriate method has been published yet [8]. Furthermore, it is still common for many pelvic surgeons to not consider uterine preservation as an effective option for prolapse treatment due to a lack of training in this kind of procedure or due to the theory that concomitant hysterectomy could have better results [9]. The aim of this review is to present the current evidence of laparoscopic options for uterus-sparing prolapse techniques, their advantages/disadvantages and their outcomes, in order to help physicians provide patients more detailed counselling and design the best therapeutic management plan for each individual.

## 2. How to Identify a Suitable Patient for Uterine Preservation

**Figure 1:**
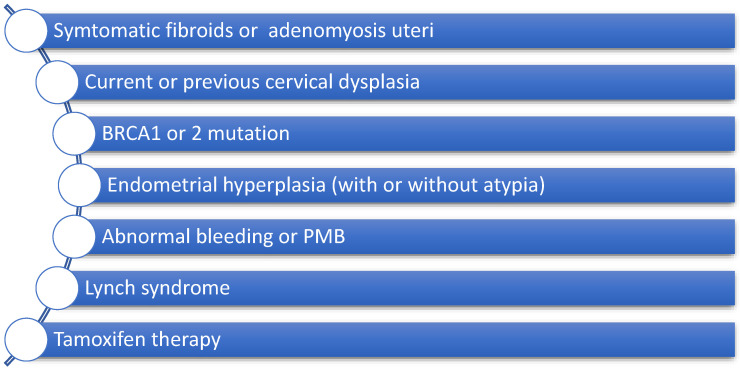
Contraindications for uterine preservation. BRCA: breast cancer gene; PMB: postmenopausal bleeding.

Performing safe and effective patient selection and counselling are crucial for planning uterus-preserving procedures for the management of POP. In order to achieve this, we must clarify the contraindications for a uterine-sparing approach [10,11,12]. First of all, patients with an elevated risk of malignancy (uterine, cervical or ovarian) should be considered an unsuitable group for performing uterine preservation [13]. The detection of genetic risk factors which predispose an individual to cancer, such as BRCA1 and 2 mutations, or human non-polyposis colorectal cancer (Lynch syndrome) are also important contraindicators for this type of procedure [14,15]. Patients diagnosed with endometrium hyperplasia with or without atypia should be excluded from hysteropexy procedures if they have a 2–25% risk of endometrial cancer [14]. Furthermore, attention must be paid in cases of women with postmenopausal bleeding (PMB), even if they have an unknown underlying uterine pathology, as unexpected endometrial malignancy or endometrium hyperplasia is actually present in 13% of negative endometrial biopsies [14]. On the other hand, POP and a normal bleeding pattern or abnormal bleeding with a negative evaluation in premenopausal patients is rarely associated with pre- cancerous or cancerous conditions, making concomitant hysterectomy in this group non-advisable as a preventive option [14]. Frick et al. also showed that obesity, which is known to be a crucial modifiable risk factor for endometrial cancer, seems to be a potential contraindication for preserving the uterus during POP management [14]. Nevertheless, women with symptomatic uterine fibroids and adenomyosis uteri or those under tamoxifen therapy are not good candidates for this kind of procedure [16]. Several studies have demonstrated that cervical elongation could be another important parameter when selecting patients for uterus-preserving procedures, as it can strongly influence the postoperative outcomes (Figure 1) [17].

Furthermore, it is really crucial to highlight the advantages and disadvantages of a uterus-sparing procedure in order to ensure the effective counselling of patients. Uterine preservation towards POP management allows patients to preserve their fertility and sense of identity. De Oliveira et al. and Meriwether et al. demonstrated that uterine-preserving techniques contribute to minimising operation times, hospital stay durations and intraoperative blood loss [18,19]. Despite the intraoperative benefits of this kind of procedure, there are also postoperative advantages which should be mentioned. In a prospective cohort study, Farquhar et al. showed that premenopausal women who underwent hysterectomy even with ovarian preservation had a two-fold higher 5-year risk of an earlier onset of menopause, which can be avoided through uterus-sparing approaches [20]. Furthermore, in a cross-sectional study, Chaudhary et al. demonstrated that undergoing a hysterectomy prior to menopause could lead to a higher incidence of menopausal symptoms and a reduced quality of life [21].

Mesh-related complications are also a vital point to be considered in selecting the best procedure for the treatment of POP. Gutman et al. highlighted in a meta-analysis that the mesh exposure led to a five-fold higher rate of complications in patients who had undergone hysterectomy combined with sacrocolpopexy (SCP) compared to those who had undergone sacrohysteropexy (SHP), with the two methods showing similar anatomical results [22]. Maher et al. showed that women who underwent SCP with concomitant total hysterectomy had a 3.5×-fold higher risk of mesh exposure in comparison with those who had undergone SHP. It is noteworthy that supracervical hysterectomy was associated with a lower risk of mesh exposure [22].

**Figure 2:**
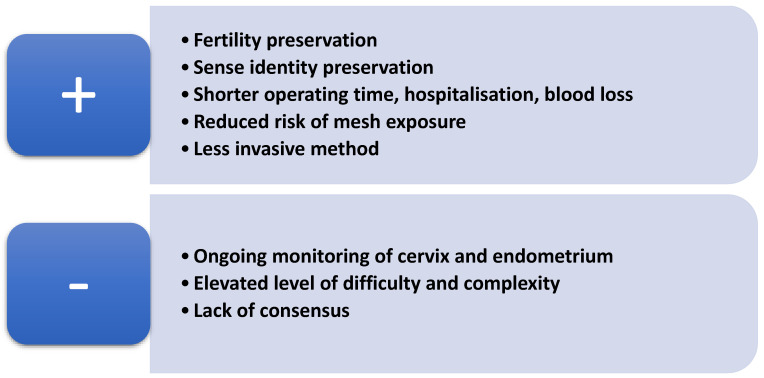
Advantages and disadvantages of uterus-preserving procedures.

On the other hand, the uterus-preserving approach seems to be accompanied by disadvantages, such as the need for ongoing cervix and endometrium monitoring [9]. Furthermore, the potentially higher difficulty and complexity of a hysteropexy procedure and potentially longer learning curves for the surgeon represent other negative aspects of these techniques (Figure 2) [23]. The lack of an established consensus for choosing the most appropriate method for hysteropexy could also be considered a disadvantage of the technique.

## 3. Available Techniques of Laparoscopic Hysteropexy

### 3.1. Laparoscopic Sacral Hysteropexy (LSHP)

This method involves the usage of a mesh which is attached to the uterus, the cervix and the anterior longitudinal ligament. However, many variations of this technique have been described in the literature according to the type of mesh used or its fixation points (anterior, posterior or even both sides of the cervix/uterus) [24]. The most commonly performed version is the method known as the Oxford technique. The procedure is mostly carried out laparoscopically, using a Y-shaped mesh, the long stem of which is attached to the anterior longitudinal ligament and its two short branches are attached to the anterior part of the cervix after passing through a window in the broad ligament [8,25]. Jefferis et al. evaluated this method in a retrospective cohort study with a 10-year follow-up and demonstrated that the Oxford technique is an effective and safe option with a low intraoperative rate of complications and a significant degree of satisfaction among patients who have undergone this procedure [26]. In a retrospective study, Sato et al. compared the composite failure (CF) of patients who had undergone LSHP and that of those who had undergone sacrocolpopexy combined with supracervical hysterectomy (LSCP/SCH) at a 24-month follow-up and demonstrated that both methods had similar results without any significant difference in CF. However, a trend suggesting a potentially statistically significant difference was detected, with the supracervical hysterectomy group having a CF rate of 10.7% and the preservation group having a rate of 3.6%. Furthermore, Sato et al. evaluated factors which could have a possible influence on the CF and showed that an elevated Ba point (anterior vaginal wall according to POP-Q Quantification System) and Body Mass Index (BMI) could be clinically relevant in regard to recurrence, despite these not having been proven to be statistically significant [27]. In a multicentre retrospective cohort study, Campagna et al. compared the intra- and postoperative results of LSHP and LSCP/SCH by collecting data on 136 patients with a 24-month follow-up, and showed that both methods were equally safe and efficient with similar anatomical outcomes and satisfaction rates among the patients. However, it was also demonstrated that LSHP seems to have a shorter operative time [28]. Nevertheless, LSHP seems to have excellent postoperative anatomic results comparable to those of total laparoscopic hysterectomy combined with sacrocolpopexy, which has resulted in statistically significant improvements in quality of life (QOL) scores [29]. In a systematic review of nine observational trials, Tius et al. showed that laparoscopic total hysterectomy or LSCP combined with SCH is associated with better objective and subjective outcomes compared to LSHP. No significant difference was observed between the two methods regarding intraoperative and postoperative complications, sexual dysfunction, postoperative stress urinary incontinence rates, recurrence rates or reoperation rates [30]. In most trials, objective success refers to improvement in POP-Q assessments, while subjective success rates refer to improvement in QOL scores. Kupelian et al. demonstrated success rates of 90%, with a follow-up period of 3 to 31.2 months, using both subjective and objective methods of evaluation [31]. Furthermore, Api et al. reported an objective success rate of 94% and subjective one of 96.3%, with a follow-up duration of 6 months [32]. An objective success rate of 98% and a subjective one of 95.2% were illustrated by Mourik et al. after a 16-month evaluation period [33]. On the other hand, Gracia et al. demonstrated a higher success rate in the group who had undergone LSCP/SCH, compared with that of the LSHP group [34].

### 3.2. Laparoscopic Uterosacral Hysteropexy (LUSH)

Several methods have been described for laparoscopic uterosacral hysteropexy (LUSH), including a plication or a shortening of uterosacral ligaments. In a retrospective study, Maher et al. showed that LUSH can be a safe and effective therapeutic option for POP with a success rate of 79% [35]. A 80% success rate from LUSH was also demonstrated by Uccella et al. [36], while Medina et al. observed a success rate of 100% [36,37]. Furthermore, Bedford et al. compared the postoperative outcomes of LUSH with those of hysterectomy combined with a laparoscopic uterosacral hysteropexy and revealed higher success rates and lower failure in the apical fixation in the group of patients who underwent hysterectomy combined with a laparoscopic uterosacral colpopexy. However, no significant difference in the need for reoperation was mentioned between either group [38]. Nevertheless, in a single centre, Haj-Yahya et al. evaluated and compared the clinical cure rates, the anatomical outcomes and the satisfaction rates of patients who had undergone LUSH with those of patients who had undergone total vaginal hysterectomy with uterosacral ligament suspension in a single-centre clinical comparative retrospective cohort study [39]. With a mean follow-up of 14.7 ± 13.23 months for the vaginal group and 17.5 ± 15.84 months for the laparoscopic group, it was demonstrated that LUSH seems to be an efficient option for women with apical prolapse who opt for techniques that involve the preservation of the uterus, offering high rates of anatomical and clinical success as well as patient satisfaction [39,40].

### 3.3. Laparoscopic Lateral Suspension (LLS)

Another surgical technique which is used for the treatment of apical prolapse is laparoscopic lateral suspension (LLS) with a mesh. This method was firstly described and carried out via laparotomy in 1967 and was further developed and adapted for laparoscopic surgery with a mesh by Dubuisson et al. [41,42]. In this method, a T-shaped mesh is used, with its central part fixated on the vesico-vaginal fascia and on the isthmus uteri and with its arms introduced retroperitoneally toward the lateral abdominal walls, alongside round ligaments, and attached to the abdominal fascia bilaterally [42,43]. Although there are many studies that investigate and evaluate the safety and efficacy of LLS with a concomitant hysterectomy, showing that it can be applied as an alternative method for POP treatment, there is a scarcity of evidence regarding the utilisation of LLS in the preservation of the uterus [44,45,46].

### 3.4. Laparoscopic Hysteropectopexy (LP)

Laparoscopic pectopexy (LP) was first described and published on in 2010 [47]. The efficacy and safety of the method was evaluated in a multicenter study, which demonstrated that LP can be applied in clinical practice as a very good alternative to laparoscopic sacropexy for apical prolapse therapy [48]. This technique can be utilised to perform hysteropexy. In this method, the central part of the mesh can be fixated to the anterior or the posterior part of the uterus according to the size of the uterus. A standard mesh can be fixated anteriorly especially in patients with small uteri. On the other hand, in patients with larger uteri, the fixation can be performed dorsally by using an extended mesh (PRP 3 × 18), avoiding causing retroflexion. In both cases, the arms of the mesh are threaded through a small opening in the broad ligament and then attached to the iliopectineal ligament (Cooper ligament) bilaterally [49]. Lee et al. compared laparoscopic hysteropectopexy (LHP) with vaginal hysterectomy in a retrospective study which included 176 patients and demonstrated that LHP seems to be more effective with regards to the length of hospitalisation, the estimated blood loss, and the average operating time, as well as the postoperative voiding difficulties [50]. However, more randomised controlled trials are needed to evaluate the clinical and anatomical outcomes of the method.

## 4. Comparison of Transvaginal and Laparoscopic Approach

Several transvaginal techniques have been described for uterus preservation in POP surgery. The Manchester procedure is the oldest one and involves cervix amputation and reattachment to the cardinal ligaments [51,52,53,54]. This technique is not preferred as it has been shown to have numerous potential complications such as dyspareunia, dysmenorrhea, infertility, and recurrent pregnancy loss as well as challenges in obtaining endometrial and cervical samples due to cervical stenosis [8,55]. In the literature, multiple modified versions of this technique are presented, which include the plication of the uterosacral ligaments posteriorly and the cardinal ligaments anteriorly in order to provide enhanced apical support [8,56]. Price et al. demonstrated that the plication of uterosacral ligaments entails an increased risk of a ureteral injury because of the difficulty in properly dissecting the ureters. Furthermore, the uterosacral ligaments are transected near the uterus, resulting in a low degree of plication [8].

Another method of transvaginal hysteropexy which has been used all over the world is sacrospinous ligament fixation. In this technique, the cervix or uterosacral ligaments are fixated to the sacrospinous ligament with a permanent or delayed absorbable suture [19]. Dietz et al. compared sacrospinous hysteropexy with vaginal hysterectomy and showed its superior efficacy and safety. However, postoperative hip discomfort and bleeding were reported in 10–15% of the cases that had undergone this method due to nerve damage involving the sacral plexus or pudendal nerve [57]. The most significant disadvantage of sacrospinous ligament fixation is the deterioration of the vaginal axis, as the fixation in this technique is carried out only unilaterally. In a Cochrane analysis of 33 RCTs, Maher et al. compared vaginal hysteropexy (especially sacrospinous hysteropexy) with sacrocolpopexy (SC) and proved that, despite its longer operating times and learning curve, SC seems to be superior to the other technique [58]. However, Hefni et al. did not observe any difference in recurrence rate and anatomical outcome between the hysterectomy and the uterus preserving-group [59]. These results were also confirmed by van Brummen et al., who highlighted the shorter recovery period after sacrospinous hysteropexy and a three-fold higher risk for overactive bladder and urge incontinence symptoms in the patients who had undergone vaginal hysterectomy in comparison with those who had undergone sacrospinous hysteropexy [60]. Van Oudheusden et al. compared in a retrospective study that included 105 patients, vaginal sacrospinous hysteropexy to LSHP with a long follow-up (4.5 years in the LSHP group and 2.5 years in the vaginal sacrospinous hysteropexy group) and showed that both methods seem to be equally effective with similar recurrence and complication rates. Operating and hospitalisation time appeared to be significantly longer in the LSHP group, while higher estimated blood loss was observed in the vaginal sacrospinous fixation group [61]. The two methods were also evaluated in a multicenter randomised controlled trial, which demonstrated that apical failure rates in both groups were equal at a 12-month follow-up. In the LSHP group, there was a higher incidence of overactive bladder (OAB) and faecal incontinence postoperatively, but a lower incidence of dyspareunia [62].

Sacrospinous hysteropexy can also be performed with the usage of a mesh, placed in the anterior vaginal wall with concomitant uterosacral ligament fixation. However, due to the United States Food and Drug Administration’s (FDA) warning in 2008 and safety communication in 2011, the utilisation of vaginal grafts has decreased significantly and sacrospinous hysteropexy is most commonly performed with the traditional method [63]. Small meshes fixed by using sutures or anchors on the uterosacral ligament are introduced have been introduced many companies in order to optimise the results of the method. However, during this approach, it can be challenging to visualise all important landmarks and anatomical structures, and most of the time, it is performed with the guidance of the index finger. Therefore, it is clear that this surgical method demands a pelvic surgeon with high expertise, as improper placement of the anchors or the sutures can lead to significant long-term discomfort for the patient [64,65]. As these data are a result of only small single-centre trials; more RCTs are needed to evaluate the method and its outcomes.

A multicentre, prospective parallel cohort study examined and compared vaginal sacrospinous hysteropexy with a mesh to LSHP at eight institutions with a one-year follow-up period and demonstrated that both methods had similar cure rates. Furthermore, estimated blood loss, complications, the duration of hospitalisation and satisfaction rates seemed to be similar for both groups. However, operating time was significantly longer in patients underwent LSHP. The vaginal mesh hysteropexy group was associated with non-significant increased mesh exposure compared to the group treated with the laparoscopic approach [66]. This cohort study was extended, with a median subjective follow-up of 7.3 ± 0.9 years, and finally revealed that both procedures had high success rates with a low incidence of complications and low reoperation rates. Although a trend indicating better anatomic results in the LSHP group was observed, the outcomes remained statistically insignificant [67]. Finally, due to the scarcity of comparative trials, conclusive results regarding optimal approach for hysteropexy are not available; therefore, there is an urgent need for well-constructed and accurately designed randomised controlled trials to be conducted in the field.

## 5. Pregnancy Outcomes After Hysteropexy

Women of reproductive age with POP who have not planned for a family should first be offered a conservative treatment option such as pelvic floor training and pessaries before opting for a surgical therapeutic option. However, in cases where a pessary does not fit well or is not desired by the patient, hysteropexy can be an alternative option. Only a limited number of studies in the literature discuss pregnancies following laparoscopic hysteropexy [10]. Bedford et al. reported one uncomplicated pregnancy in a hysteropexy group, resulting in a caesarean section (C-section), without any evidence of postpartum prolapse recurrence [38]. Another study involving 43 women who underwent laparoscopic hysteropexy reported two successful pregnancies with sufficient pelvic floor support in all three compartments [35]. Furthermore, in a small retrospective study, Seracchioli et al. investigated 15 women of reproductive age with genital prolapse who underwent hysteropexy. Three pregnancies were reported postoperatively, out of which one resulted in a miscarriage, and the other two had completed pregnancy terms, with delivery by caesarean section. The authors mentioned that none of the patients experienced prolapse recurrence or required a reoperation [68]. Jefferis et al. reported a cases series of 6 patients with spontaneous conception after undergoing an Oxford hysteropexy and showed that foetal growth and uterine support are not affected by the technique. In this small case series, two patients were detected with de novo anterior vaginal prolapse postpartum [69]. Although pregnancies do not seem to influence the outcome of prolapse surgery, the number of pregnancies reported after hysteropexy is really limited. Multicenter randomised controlled trials are needed in order to evaluate the influence of a future pregnancy on the outcome of laparoscopic hysteropexy. It is crucial to investigate which type of laparoscopic hysteropexy is the most appropriate for each patient undergoing hysteropexy without any plans to start a family as well as to identify the adequate delivery mode. Given that relatively few women undergo hysteropexy followed by pregnancy, designing a feasible study to address these questions would be challenging.

## 6. Conclusions and Future Perspectives

Nowadays, there is a growing trend among women to opt for POP surgery and uterus preservation. It is widely accepted that hysterectomy itself does not contribute significantly to the treatment of pelvic floor defects. In contrast, it has been shown that concomitant hysterectomy results in longer operating times and a higher incidence of mesh exposure when total hysterectomy is carried out. It is really crucial for pelvic surgeons to understand the indications for a concomitant hysterectomy in order to perform appropriate counselling of patients who opt to preserve their uteri. Although various hysteropexy procedures (abdominal, laparoscopic, vaginal) have been described in the literature, there is still no consensus on the appropriate method in different cases. More randomised multicentred controlled trials are needed for the optimal evaluation and comparison of the methods.

## Data Availability

As this is a review article, no data were created.
